# Using mixed reality technique combines multimodal imaging signatures to adjuvant glioma photodynamic therapy

**DOI:** 10.3389/fmed.2023.1171819

**Published:** 2023-07-18

**Authors:** Jiawei Dong, Fang Wang, Yuyun Xu, Xin Gao, Hongtao Zhao, Jiheng Zhang, Nan Wang, Zhihui Liu, Xiuwei Yan, Jiaqi Jin, Hang Ji, Ruiqi Cheng, Lihai Wang, Zhaowen Qiu, Shaoshan Hu

**Affiliations:** ^1^Department of Neurosurgery, The Second Affiliated Hospital of Harbin Medical University, Harbin, China; ^2^Cancer Center, Department of Neurosurgery, Zhejiang Provincial People’s Hospital, Affiliated People’s Hospital, Hangzhou Medical College, Hangzhou, Zhejiang, China; ^3^Cancer Center, Department of Radiology, Zhejiang Provincial People's Hospital, Affiliated People's Hospital, Hangzhou Medical College, Hangzhou, Zhejiang, China; ^4^Heilongjiang Tuomeng Technology Co., Ltd, Harbin, China; ^5^College of Engineering and Technology, Northeast Forestry University, Harbin, China; ^6^College of Information and Computer Engineering, Northeast Forestry University, Harbin, China

**Keywords:** glioma, photodynamic therapy, magnetic resonance imaging, mixed reality, HoloLens

## Abstract

**Background:**

Photodynamic therapy (PDT) promotes significant tumor regression and extends the lifetime of patients. The actual operation of PDT often relies on the subjective judgment of experienced neurosurgeons. Patients can benefit more from precisely targeting PDT’s key operating zones.

**Methods:**

We used magnetic resonance imaging scans and created 3D digital models of patient anatomy. Multiple images are aligned and merged in STL format. Neurosurgeons use HoloLens to import reconstructions and assist in PDT execution. Also, immunohistochemistry was used to explore the association of hyperperfusion sites in PDT of glioma with patient survival.

**Results:**

We constructed satisfactory 3D visualization of glioma models and accurately localized the hyperperfused areas of the tumor. Tumor tissue taken in these areas was rich in CD31, VEGFA and EGFR that were associated with poor prognosis in glioma patients. We report the first study using MR technology combined with PDT in the treatment of glioma. Based on this model, neurosurgeons can focus PDT on the hyperperfused area of the glioma. A direct benefit was expected for the patients in this treatment.

**Conclusion:**

Using the Mixed Reality technique combines multimodal imaging signatures to adjuvant glioma PDT can better exploit the vascular sealing effect of PDT on glioma.

## 1. Introduction

Adult glioma is the most common central nervous system tumor (CNS). Over the last 20 years, many studies have uncovered molecules and signaling pathways. However, surgical treatment combines with radiotherapy or chemotherapy is still the principal treatment of malignant gliomas (1). According to the 2016 World Health Organization (WHO) classification of tumors of the CNS, the prognosis of patients with glioblastoma (GBM) in China remains dismal (2, 3). The extent of resection is well established prognostic factor for gliomas (4). Most malignant gliomas recur within 2 cm of the original resection site (5). Distinguishing the tumor boundary clearly and removing the glioma to the maximum to protect the function is a significant problem we must confront.

Magnetic resonance imaging (MRI) is integral in the noninvasive diagnosis and therapeutic monitoring of glioma. Because gliomas extensively infiltrate the surrounding normal brain, maximum resection of T1-weighted contrast-enhanced magnetic resonance imaging has been consistently associated with more remarkable survival (6, 7). The blood–brain barrier is often disrupted in glioma patients, T1-weighted post-contrast enhancement (T1W + C) can show these areas (8). There are still invasive glioma cells and new capillaries in the tumor infiltrative boundaries, which may be the root of tumor recurrence (9). Perfusion-weighted imaging (PWI) is a hot research topic for many tumors in China. Dynamic susceptibility contrast MRI (DSC-MRI) is widely used to evaluate the distribution of microcirculation and blood perfusion (10). Among the PWI findings, Cerebral blood flow (CBF) can be quantified in absolute values (ml/min/100 g brain tissue) (11). Recently, it was shown that CBF obtained by PWI has a positive correlation with micro-vessel density (12). In parallel, CBF correlated with VEGF expression in glioma and was an independent risk factor for OS (13).

Photodynamic therapy (PDT) has emerged as a potent treatment against tumors. In addition to generating highly toxic reactive oxidative species (ROS), the indirect induction of vascular shutdown is not negligible (14). Dermatologists and gastroenterologists have been treating intractable diseases based on the sealing effect of PDT on the vascular system (15, 16). The conventional PDT surgery-based protocol involves extensive tumor bed irradiation after resectioning the glioma (17). It is beneficial but still not enough for glioma patients. Understanding the morphological characteristics of gliomas is essential to find an important target region for PDT.

Experienced neurosurgeons will focus on the critical areas during the procedure. Much of this experience can be understood but cannot be described accurately. The advent of virtual reality simulation has ushered forward an era. Mixed reality (MR) is a new technology that can provide an alternative to Virtual Reality (VR) (18). Compared to VR, MR extends virtual technology to reality and allows interaction modes (19). Microsoft HoloLens (20) was developed by Microsoft and enabled people to describe an environment in which virtual and real elements overlap. It is used in various applications and allows users to interact with their surroundings using a hologram. Thoracic and spine surgery were early adopters of MR technology and had been widely used in clinical practice (21, 22). This study aims to develop an advanced method based on HoloLens in the medical field. We focus on the application scenarios and feasibility of MR In the glioma PDT. This technique may change the conventional glioma PDT protocol and has a high potential for popularization.

## 2. Methods

### 2.1. Data acquisition

Our Institutional Review Board approved this study. These cases were completed with a team approach, including one chief neurosurgeon, 2 attending neurosurgeons, and one radiologist who participated in these procedures. Ten patients (5 female), including primary and recurrent gliomas (WHOII-IV), between February 2022 and June 2022. Patients were not purposely selected; the numbers are in the order of their visits during this period. All patients provided informed consent. The average age of the patients was 55.1 ± 10.5. Patient clinicopathological characteristics are listed in Table 1. At the end of the follow-up, no patients were dead. TCGA data analysis Survival data for glioma patients has acquired from The Cancer Genome Atlas (TCGA) database.^1^ A Cox proportional hazards regression model was established using the Coxph (23) function of the R package survival (version 3.2–7) to analyze the relationship between gene expression and prognosis in gliomas. A statistical test using the Logrank test was performed to obtain prognostic significance.

**Table 1:** Patient demographics and glioma pathology.

Patient	Sex	Age	Glioma pathology (WHO)
1	Female	53	WHO IV
2	Female	47	WHO IV
3	Male	55	WHO III
4	Female	59	WHO IV
5	Female	51	WHO III
6	Male	54	WHO IV
7	Male	68	WHO IV
8	Male	55	WHO III
9	Male	34	WHO II
10	Female	75	WHO IV

### 2.2. Preoperative MRI protocols

All patients were examined with two 3.0-T MRI systems (Signa; General Electric Medical System, USA, and Skyra; SIEMENS Medical System, DEU). The examination included standard pulse sequences for brain tumors (axial T1WI, T2WI, T2 fluid-attenuated inversion recovery T2-FLAIR, DTI, gadolinium-enhanced T1-weighted sequences, and PWI MRI). The DTI sequence was acquired with the following parameters: repetition time/echo time (msec), 8637/64.1; acquisition matrix 128, flip angle 90, pixel spacing 0.75/0.75, bits allocated 16. The PWI sequence was acquired with the followings: slice thickness 4, repetition time/echo time (msec) 2,110/30, imaging frequency 123.25, spacing between slices 5.2, acquisition matrix 128, flip angle 90, pixel spacing 1.71/1.71. Patient image data were finally stored in DICOM 3.0 format.

### 2.3. 3-D model construction

3D digital reconstructions were created based on patients’ anatomy before their planned surgery. Patient MRI data was registered on the cloud service.^2^ The image registration and 3D visualization used the Tuoying system®. Imaging experts edited the 3-D reconstructions to enhance visualization by assigning particular color and opacity values to voxel ranges. The processed data was finally stored in stereolithography (STL) format and downloaded using HoloLens glasses. Attending neurosurgeons and radiologists oversaw the production of these models. Table 2 shows respondents’ evaluation of the accuracy of the visual model, which was assessed through four statements using a 5-point Likert scale (degree from “strongly agree” to “strongly disagree”).

**Table 2:** The questions and scoring of a 5-point Likert scale (1 = very poor, 5 = excellent).

Question no.	Questionnaire	Strongly disagree	Disagree	Neutral	Agree	Strongly agree
		1	2	3	4	5
1	Were tumor of this 3D model similar to real tumor tissue	–	–	–	–	–
2	Whether this 3D model can be matched to the same perspective as the real patient?	–	–	–	–	–
3	Whether this 3D model reflects the tissue with adequate blood supply area in the peritumoral brain zone?	–	–	–	–	–
4	Whether this HoloLens software system can simulate the situation of brain tissue after tumor resection?	–	–	–	–	–

### 2.4. Immunohistochemistry

Glioma tissues were formalin-fixed, paraffin-embedded, and sectioned at a thickness of 4 μm. Immune complexes were detected with the SP Kit (Solarbio, Beijing, China) and DAB Substrate Kit (Solarbio, Beijing, China). Signals were detected using Olympus BX41 microscope. Immunohistochemistry was performed using standard protocols as described previously (24). Quantification of Immunohistochemistry (IHC) staining was performed in a blinded fashion. ImageJ and ImageJ plugin IHC profiler was applied to quantify IHC staining analysis as reported (25).

### 2.5. Photo dynamic therapy

Patients received the Hematoporphyrin Injection (5 mg/kg) intravenously 48 h before surgery and were strictly protected from light after administration. An experienced neurosurgery professor performed the craniotomy. The same professor performed the PDT after the removal of the tumor. PDT light sources used 630 nm in the red region. The laser power density was referenced to the China Anti-Cancer Association’s technical guidelines and conformed to international operational standards (26, 27). The average power density was 100 mW/cm^2,^ while hyperperfused areas were irradiated with 150–200 mW/cm^2^ power density.

### 2.6. Statistical analysis

**Figure 1:**
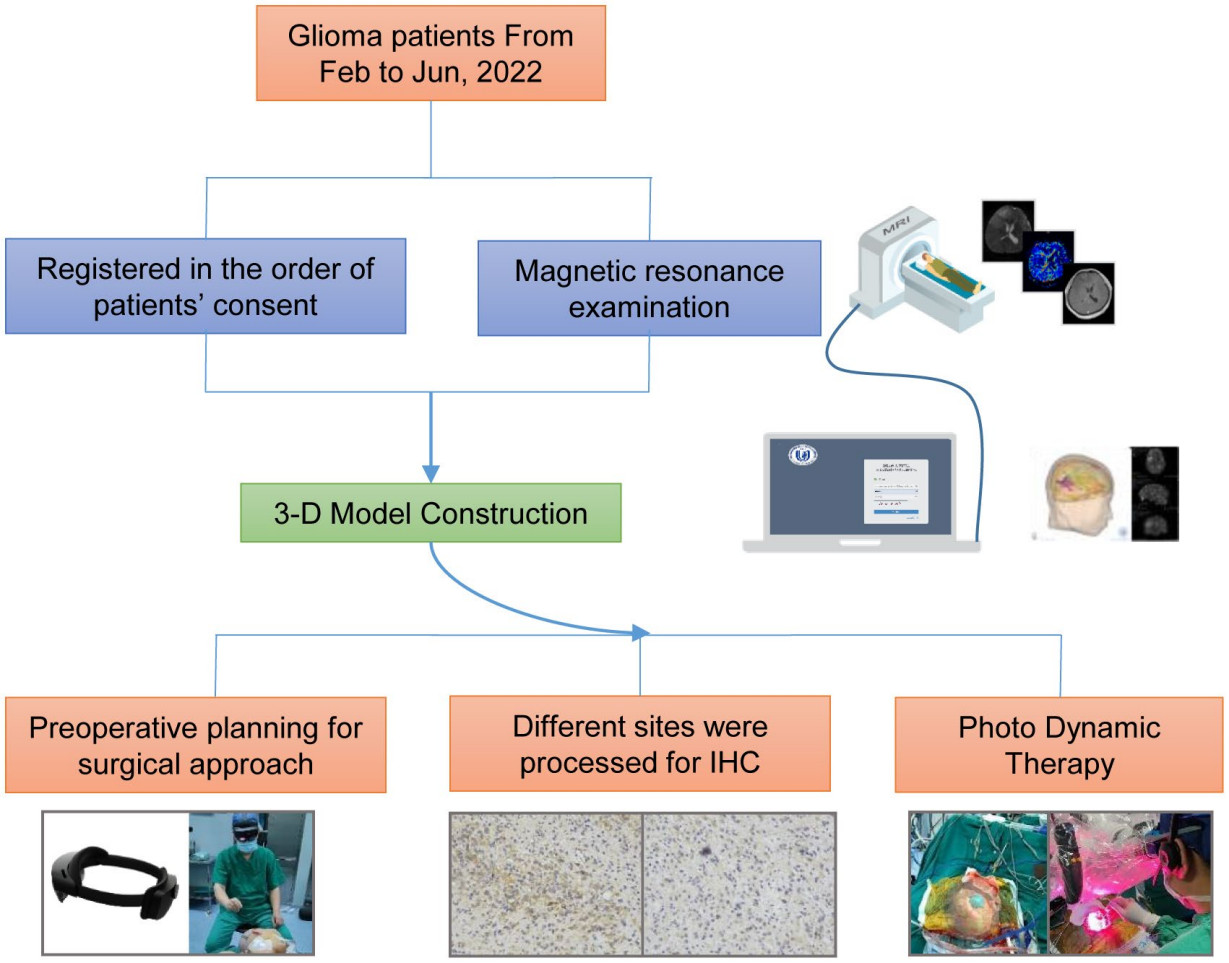
A flowchart of the entire procedure.

IHC data were analyzed using an unpaired Students t-test using GraphPad/Prism 9.0. Cox-Regression Survival analysis was performed using the R package survival (version 3.2–7), and survival data were compared using a Logrank test. *p* < 0.05 was considered significant at 0.05 level (*p < 0.05, **p < 0.01, ***p < 0.001). The experimental flow chart was shown in Figure 1.

## 3. Results

### 3.1. Intraoperative imaging showed different blood supply in different areas of glioma

**Figure 2:**
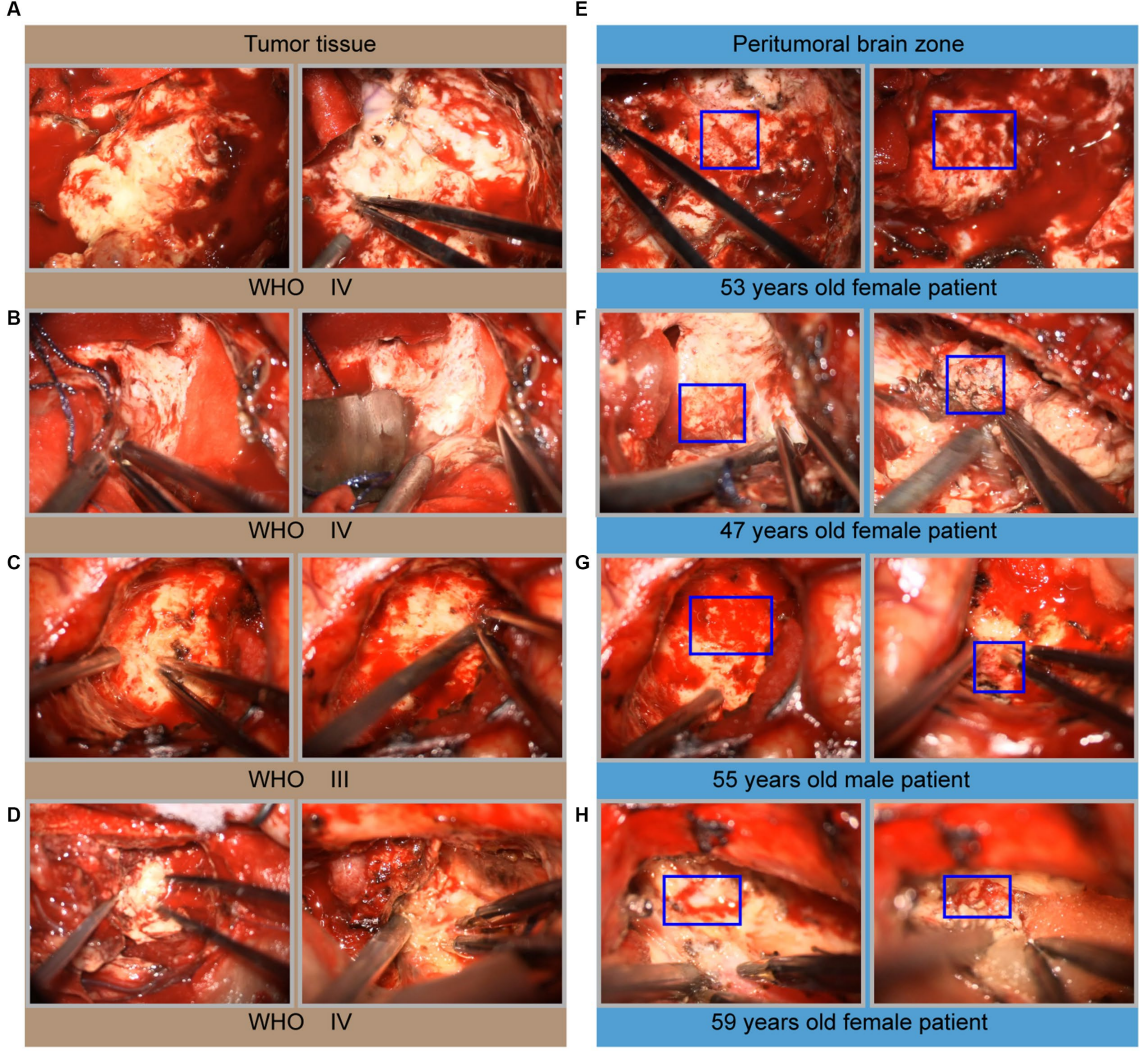
Microscopic intraoperative view of glioma. **(A–D)** Microscopic images of solid tumor tissue, including one grade III glioma and four grade IV gliomas. **(E–H)** Microscopic images of the peritumoral brain zone corresponding to the same patient. The hemorrhage areas seen intraoperatively were marked with a blue box in **(E–H)**.

During glioma surgical resection, we found that the glioma tissue mass was grayish-white, and the tumor texture was harder than the surrounding healthy brain tissue (Figures 2A–D). Interestingly, hemorrhage of glioma capillaries was noted in multiple areas as the operation proceeded. These areas of hemorrhage occur more in the periphery of the glioma, with few capillaries penetrating the center (Figures 2E–H). These findings suggest that some sites may provide significant blood perfusion for the oxygen and nutrients needed for further glioma growth.

### 3.2. T1WI + C and PWI of glioma

**Figure 3:**
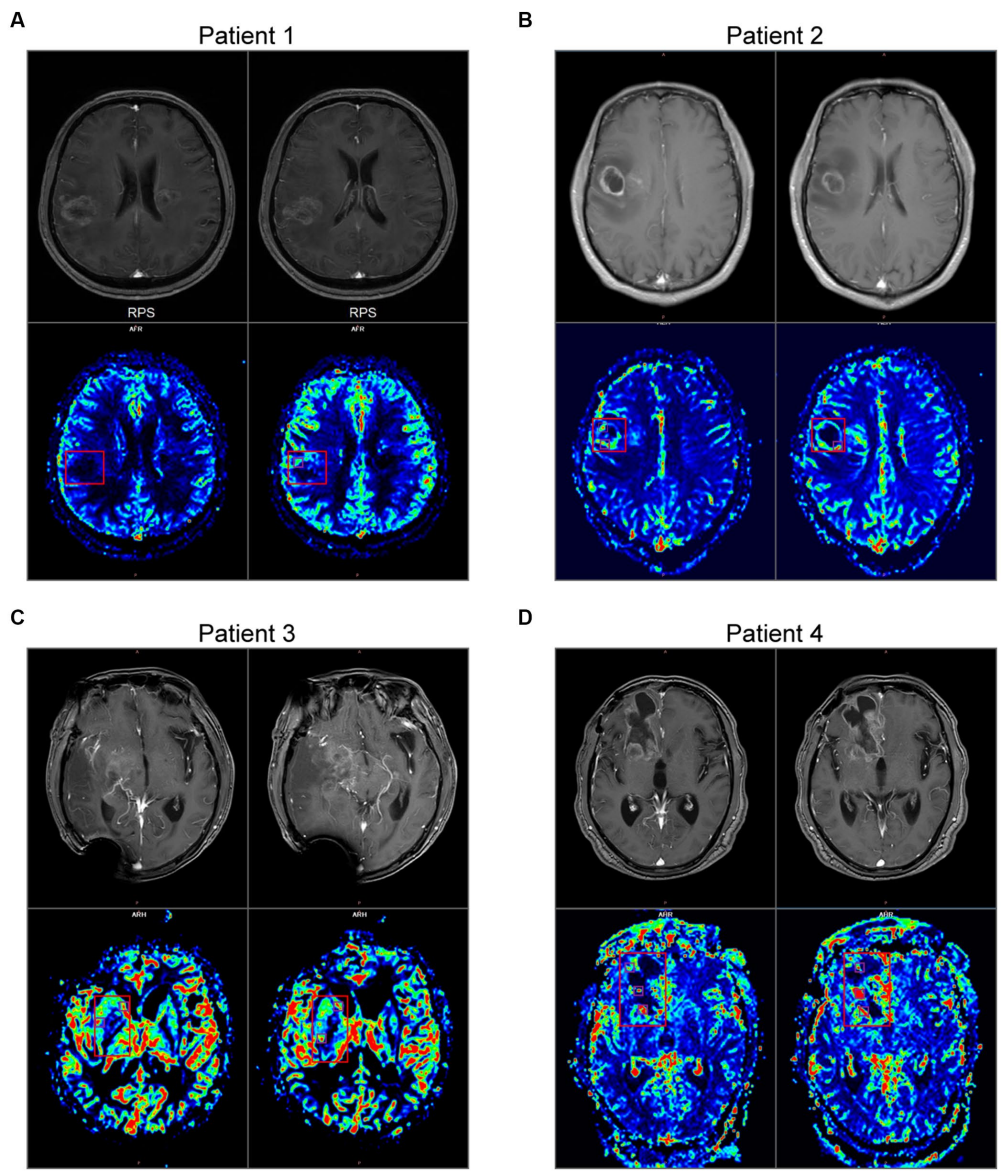
MRI and PWI images of 4 gliomas. **(A,B)** Imaging characteristics of primary glioma. Upper: the images of T1W + C. Bottom: the images of CBF in PWI. **(C,D)** Imaging characteristics of recurrent glioma. The main body of the glioma is marked with a red box, and the areas of relatively high CBF values at the peritumoral brain zone are marked with purple boxes.

Next, to analyze these crucial areas of hemorrhage, all newly admitted glioma patients underwent T1WI + C and PWI on admission in our center. Here we took two primary glioma cases and two recurrent glioma cases as examples to investigate the clinical significance. The primary glioma margins showed an irregularly contrast-enhancing shape. Compared with the images of T1W + C, we found that the PWI in the marginal region also exhibited various CBF values (Figures 3A,B). One or more areas of relatively high CBF values could be seen at the tumor margins. In contrast, these imaging features were also present in recurrent gliomas, and the presentation was more complex (Figures 3C,D). This founding might be related to the recurrence of glioma at multiple places.

### 3.3. 3-D reconstruction of DTI and glioma

**Figure 4:**
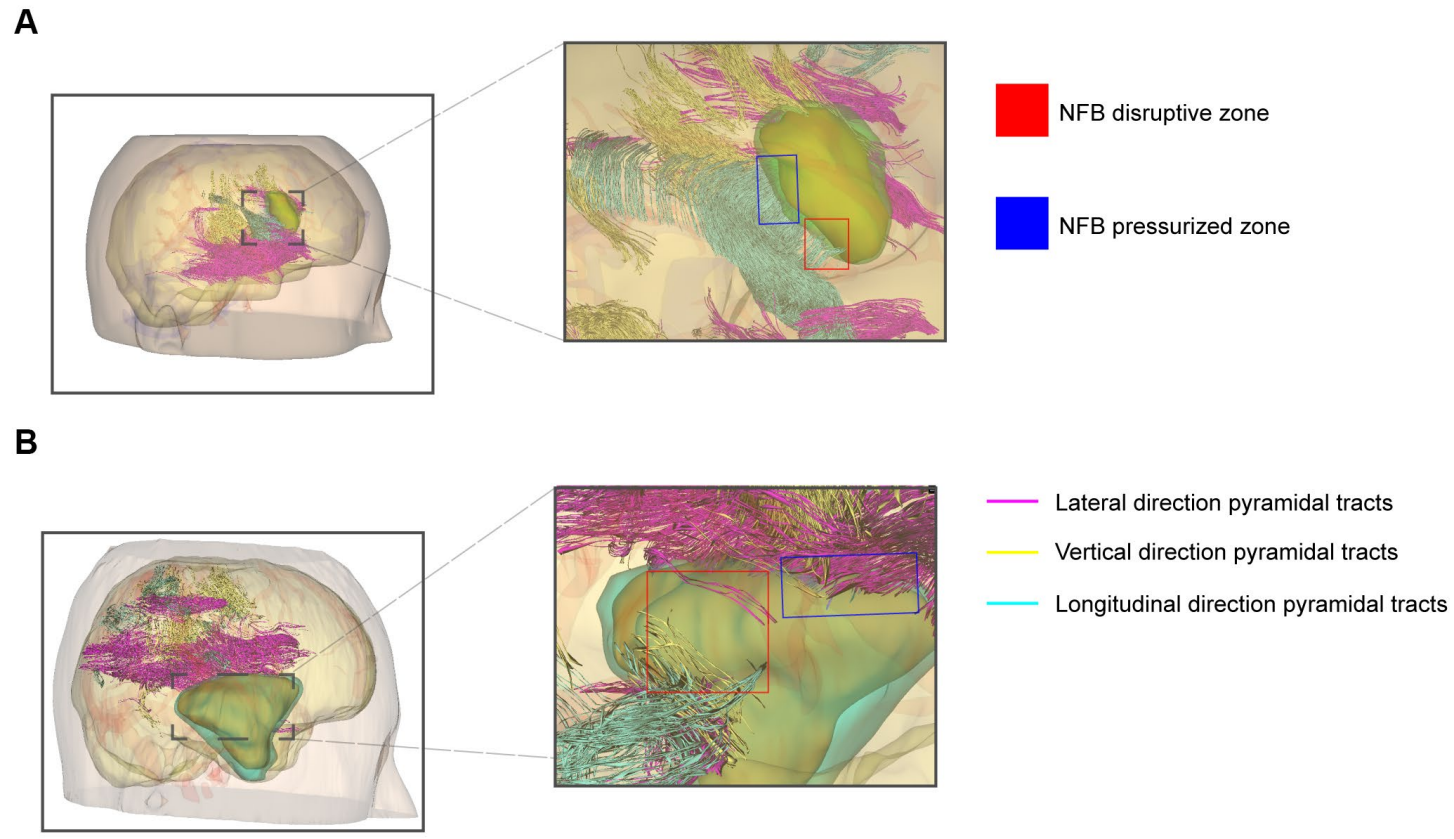
3-D visualization reconstruction of pyramidal tracts and glioma. **(A)** Reconstructed image of primary glioma. **(B)** Reconstructed image of recurrent glioma. Red boxes indicate the nerve fiber bundles disruptive zone; blue boxes indicate NFB pressurized zone. The purple, yellow, and cyan lines indicate the pyramidal tracts traveling in different directions in the reconstruction.

Neurosurgeons use the Tuoying system® to reconstruct MRI images, and imaging specialists re-plan and reconstruct gliomas with an unclear boundary. To visually and intuitively demonstrate the effects of glioma on pyramidal tracts, we performed a 3-D reconstruction of the patient’s DTI and T1W + C data. Three colors were used to distinguish the DTI-derived nerve fiber bundles. 3-D visualization of DTI data allowed different perspectives on the relationship between pyramidal tracts and gliomas. We found nerve fiber bundles were pushed or destroyed in different areas of the tumor margin obtained by observing the 3D model (Figures 4A,B). The vigorous growth of the tumor may cause the formation of these destroyed areas.

### 3.4. Combine multiple modal MR images and 3-D visualization

**Figure 5:**
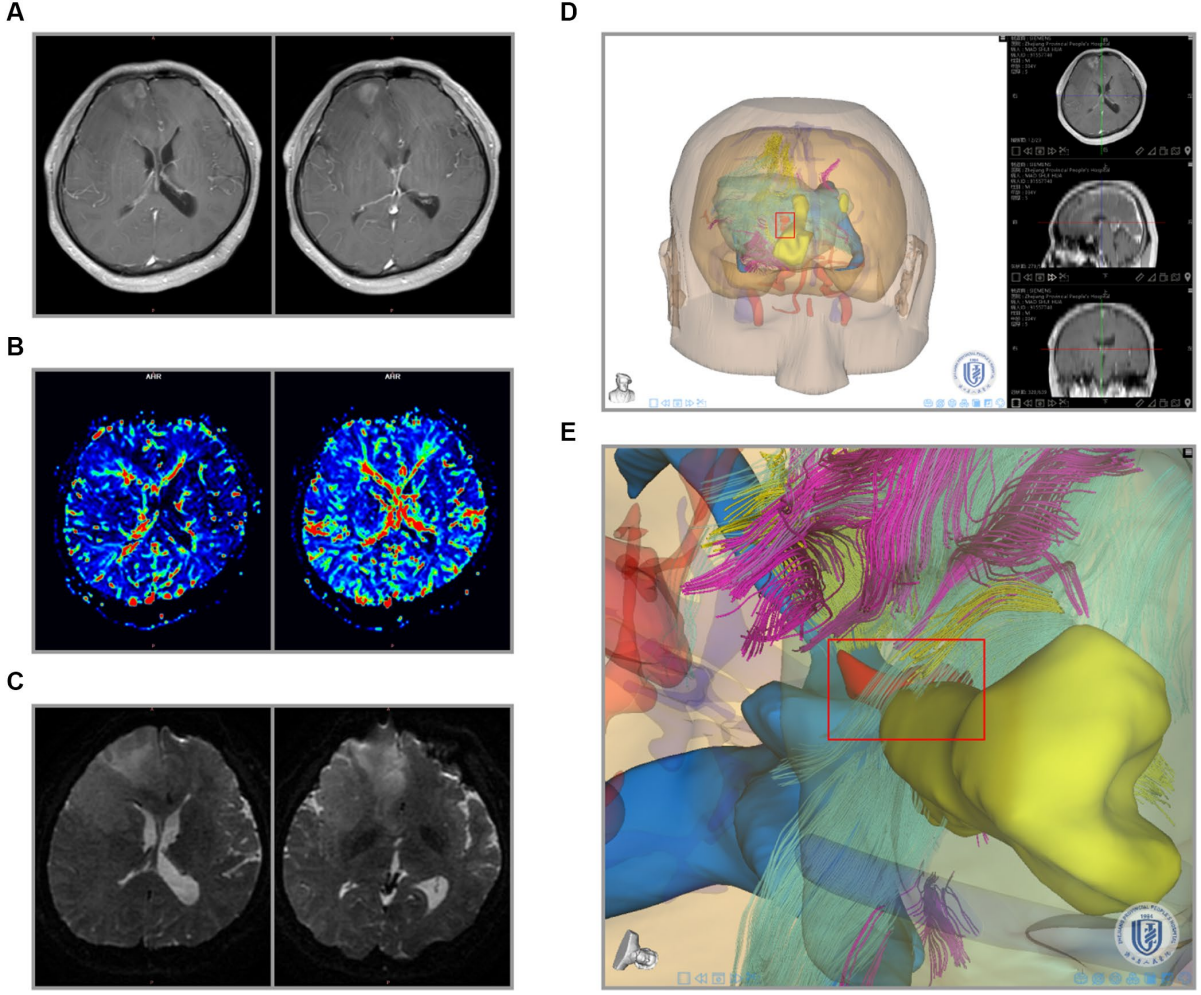
Integration of primary glioma MRI data and 3-D reconstruction. **(A)** Preoperative radiological findings (T1W + C) of glioma. **(B)** PWI cerebral blood flow (CBF) of the glioma. **(C)** DTI nerve appearance of glioma patients. **(D)** 3-D visualization reconstruction of all MRI images using the Tuoying system, and **(E)** is a locally enlarged drawing of **(D)**.

**Figure 6:**
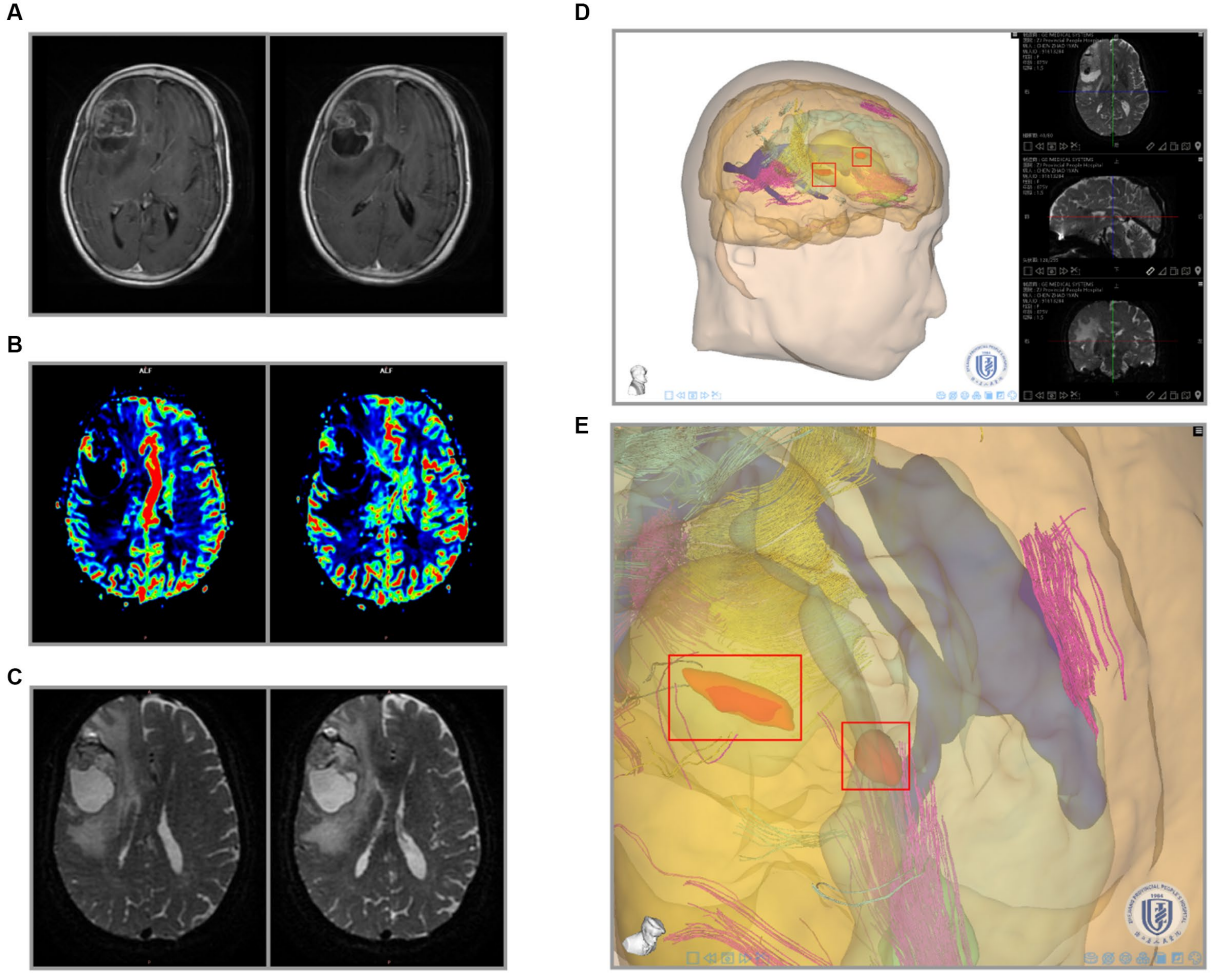
Integration of recurrent glioma MRI data and 3-D reconstruction. **(A)** Preoperative radiological findings (T1W + C) of glioma. **(B)** PWI cerebral blood flow (CBF) of the glioma. **(C)** DTI nerve appearance of patients. **(D)** 3-D visualization reconstruction of all MRI images using the Tuoying system, and **(E)** is a locally enlarged drawing of **(D)**.

The above results revealed that the peritumoral gliomas had specific areas affecting blood supply and nerve fiber tracts. To further analyze the association between these data, we tried 3-D image registration and combined T1WI + C, CBF, and DTI images together (Figures 5A–D, 6A-D). The 3-D view showed hyperperfusion in part of the glioma areas, while the places with a rich blood supply were accompanied by nerve fiber bundles destroyed (Figures 5E, 6E). These cases demonstrate that we provided an intuitive preoperative visualization for these focal regions. Meanwhile, the discovery and localization of these specific regions may offer a guiding suggestion for further manipulation after surgical resection of the glioma subject.

### 3.5. Mixed reality technique combined glioma PDT workflow

**Figure 7:**
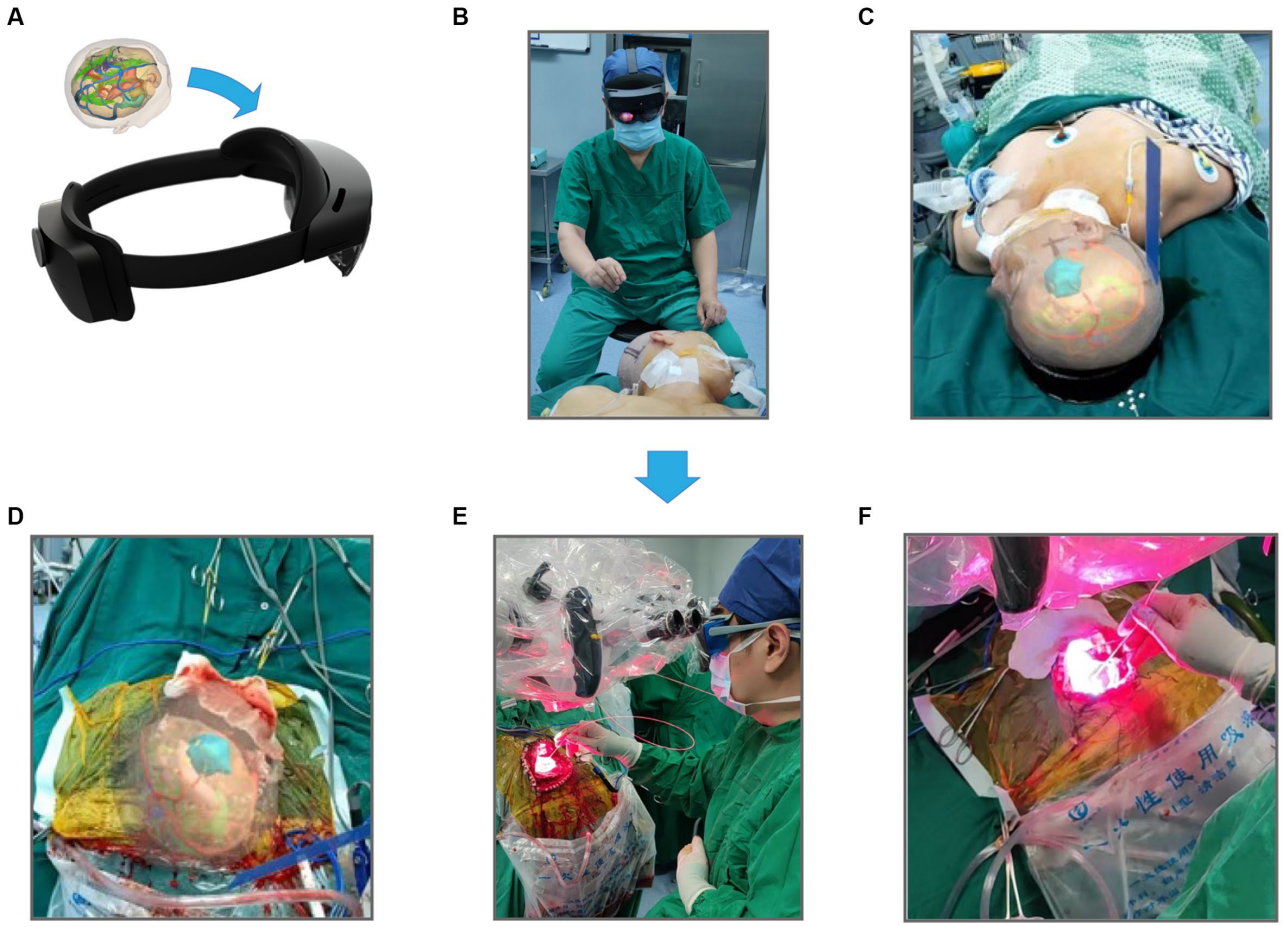
The workflow of mixed reality technique combined PDT. **(A)** Store 3-D reconstructed images using STL format and import them into HoloLens glasses. **(B)** Preoperative selection of the best surgical position for craniotomy based on reconstruction results. **(C)** Images are seen by the operator wearing HoloLens glasses. **(D)** Intraoperative resection of the main body of the glioma, the actual extent of resection was compared with the extent of preoperative reconstruction. **(E)** Irradiation treatment of glioma margins using PDT, and **(F)** is an enlarged drawing of **(D)**.

We converted these data to an STL file to apply the above results to PDT and registered them in our remote server (Figure 7A). The 3D virtual brain interaction image was superimposed onto the patient’s head through HoloLens. The surgical position is a crucial part of craniocerebral surgery, and neurosurgeons can perform preoperative planning by interacting with the object (Figures 7B,C). Our system provided gesture instructions that guided the user to view the space after the simulated removal of the glioma (Figure 7D). After that, the tumor bed was treated with standard-fluence PDT (Figure 7E). Meanwhile, high-dose PDT was applied to the high-perfusion areas located before surgery under the premise of no brain tissue burning (Figure 7F). The advantage of performing PDT in this gradient was that it protected normal brain tissue while preventing glioma recurrence.

### 3.6. Tissue specimens at different sampling sites show different expressions of pro-angiogenic factors

**Figure 8:**
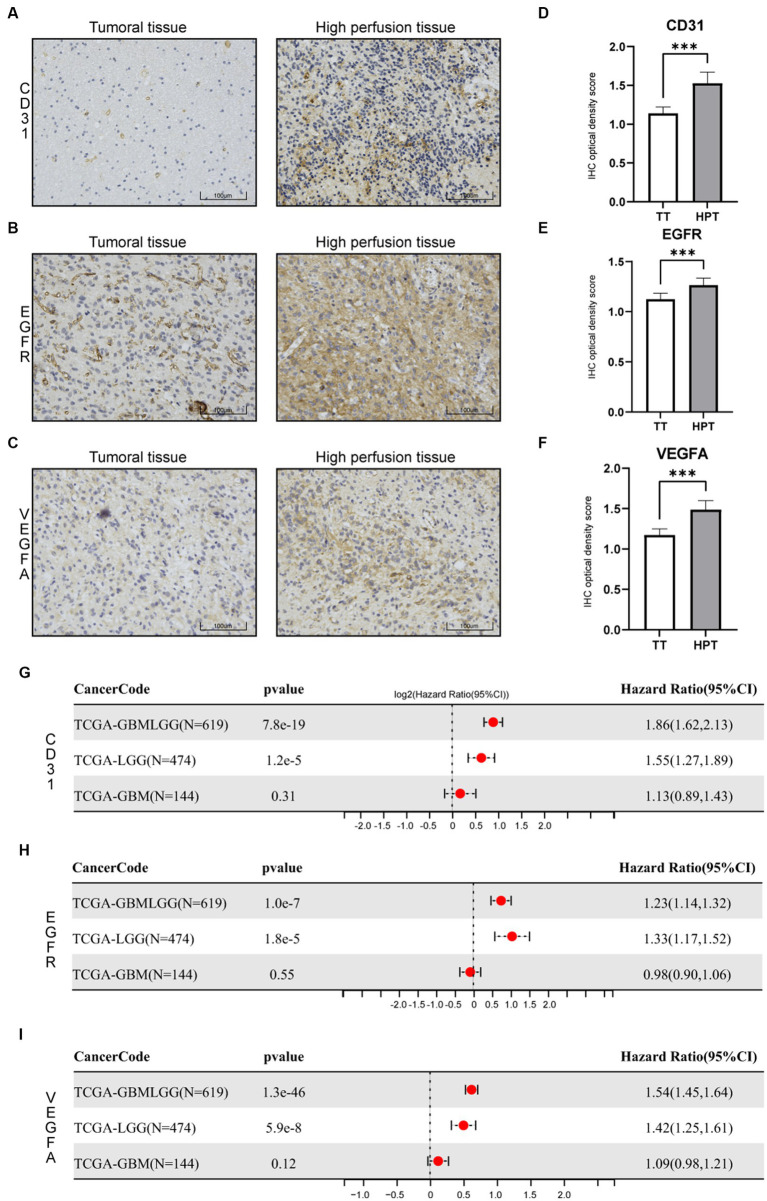
Immunohistochemistry (IHC) of tissue specimens at different sampling sites. **(A)** CD31 IHC. **(B)** EGFR IHC. **(C)** VEGFA IHC. **(D–F)** Statistical evaluation of the IHC results. *p < 0.05, **p < 0.01, and ***p < 0.001. **(G–I)** Cox proportional hazards regression model of three genes in glioma(LGGs, GBM, and all glioma).

For the validation, the glioma samples were carried out on three GBM patients by one chief neurosurgeon based on preoperative localization. CD31, EGFR, and VEGFA expression were examined. We carried out immunohistochemical staining of the CD31, EGFR, and VEGFA proteins in primary glioma of tumor sites and hyperperfusion sites. We found that the expression patterns of CD31, EGFR, and VEGFA, which are related to angiogenesis, are strikingly different in different sites (Figures 8A–C). We determined protein levels using a 4-step grading system (0, 1, 2, and 3 for negative, weak, strong, and extreme staining, respectively). Three angiogenesis factors expressions were high in the hyperperfusion sites corresponding to hemorrhage areas during the operation (Figures 8D–F). Finally, we examined whether CD31, EGFR, and VEGFA expression were associated with glioma’s prognosis in the TCGA dataset. The Cox proportional hazards regression model was implemented in the R survival package. The Logrank test was used to determine the statistical significance of the survival. We observed high CD31, EGFR, and VEGFA expression predicted poor prognosis in glioma patients (Figures 8G–I).

## 4. Discussion

Mixed reality (MR) has gained popularity in the recent decade. In this work, a sophisticated mixed-reality solution for neurosurgery has been presented. Based on the advantages of PDT, we developed a surgical model that targets glioma hyperperfusion sites. With MR system-assisted localization, this surgical model is feasible and reliable in the PDT of glioma. More importantly, with many of these experiences, other types of intracranial surgery can also be used, such as intracerebral hemorrhage, cerebral aneurysms, and endoscopic skull base surgery. Our protocol of fusing multiple MRI data provided a potential example for the preoperative planning of other tumors.

Although surgical resection of gliomas is becoming increasingly sophisticated, many neurosurgical experts believe a complete surgical resection of gliomas is virtually impossible. The malignant process and recurrence of gliomas are characterized by neoangiogenesis (28). Thus, accurate assessment of neovascularization is crucial for grading and therapeutic. PWI is a helpful scan mode in glioma grading by detecting vascular density (29). Several studies have elucidated that some molecules may be involved in the angiogenic regulation of gliomas. Direct impairment of tumor microvasculature has an antitumor effect (30). Vascular Endothelial Growth Factor (VEGF) has become a significant target of anti-glioma therapy (31). Platelet endothelial cell adhesion molecule 1 (PECAM-1/CD31) is involved in microvascular proliferation, and EGFR signaling is required for angiogenesis (32–34). Early studies have shown that PDT causes microvascular dysfunction and reduced growth in gliomas (35, 36). Along these lines, our team chose to perform gradient irradiation on the tumor bed and focus on the area of hyperperfusion sites during the process of PDT. Experienced neurosurgeons often perform this PDT regimen, and the exact location of irradiation is challenging to describe. Although 3-D reconstruction has been widely used in surgery, our MR system provides a new 3-D tumor morphology that visualizes the integration of tumor, nerve and blood flow for both the operator and the learner. In addition, due to the layered tissue reconstruction, we have the advantage of being able to hide skin, bone, and other tissues to see the tumor area more clearly.

Glioma has unclear boundaries with surrounding tissues. 3-D reconstruction required repeated corrections by professional imaging specialists, which took much time. Perhaps we can introduce AI technology to complete the reconstruction work quickly. Several neurosurgeons evaluated this model accurately (measured on a 5-point Likert scale in Supplementary Figure S1). Unfortunately, the brain tissue will inevitably shift with the increase in the scope of the tumor resection and the redistribution of intracranial pressure (37). It can have a fatal effect on operations that rely too heavily on preoperative localization. These variables should be the focus of our following research and development to make this visual model more similar to imaging-navigation surgery. At the moment, HoloLens relies on the doctor’s hand gestures, often leading to conflicts with surgical instruments. As of the submission, there were no deaths in the enrolled patients. We will continue to follow these patients and supplement the control group in subsequent studies. Finally, although this study showed beneficial trends for both neurosurgeons and patients, we could not quantify this advantage due to the small sample size.

Mixed reality visualizations are rapidly integrating into medical operations and clinical teaching. With the improvement of MRI, we have made certain breakthroughs in the 2-D level of glioma. We incorporated various details of glioma obtained from MRI into a visual model and accumulated some experience. This model provided more referenceable information to the surgeon during surgery. This study focused on the tumor blood supply for PDT. Applying this image fusion technique can concentrate on areas of interest to other specialties. Our study can provide a new surgical idea for other craniocerebral diseases and even other systemic diseases in the future.

## Data availability statement

The original contributions presented in the study are included in the article/Supplementary material, further inquiries can be directed to the corresponding authors.

## Ethics statement

The studies involving human participants were reviewed and approved by the Animal Ethical and Welfare Committee of Zhejiang Provincial People’s Hospital. The patients/participants provided their written informed consent to participate in this study. Written informed consent was obtained from the individual(s) for the publication of any potentially identifiable images or data included in this article.

## Author contributions

SH, JD, and ZQ conceived and designed the study, and revised the manuscript. JD, FW, YX, XG, and XY provided analytical technical support. ZL, NW, JZ, and LW participated in the production of charts and pictures. HZ and RC revised the manuscript. SH supervised the study and performed the surgery as a chief neurosurgeon. All authors have read and approved the final manuscript.

## Funding

This work was funded by the National Natural Science Foundation of China (No. 61575058).

## Conflict of interest

RC was employed by the company Heilongjiang Tuomeng Technology Co., Ltd.

The remaining authors declare that the research was conducted in the absence of any commercial or financial relationships that could be construed as a potential conflict of interest.

## Publisher’s note

All claims expressed in this article are solely those of the authors and do not necessarily represent those of their affiliated organizations, or those of the publisher, the editors and the reviewers. Any product that may be evaluated in this article, or claim that may be made by its manufacturer, is not guaranteed or endorsed by the publisher.

## Footnotes

^1^https://tcga-data.nci.nih.gov/tcga/

^2^http://8.142.138.102/zjph
