# Supplementary figures and images for: Using mixed reality technique combines multimodal imaging signatures to adjuvant glioma photodynamic therapy
Source: Front Med (Lausanne). 2023 Jul 18;10:1171819. doi: 10.3389/fmed.2023.1171819 (PMC10392826; doi:10.3389/fmed.2023.1171819)

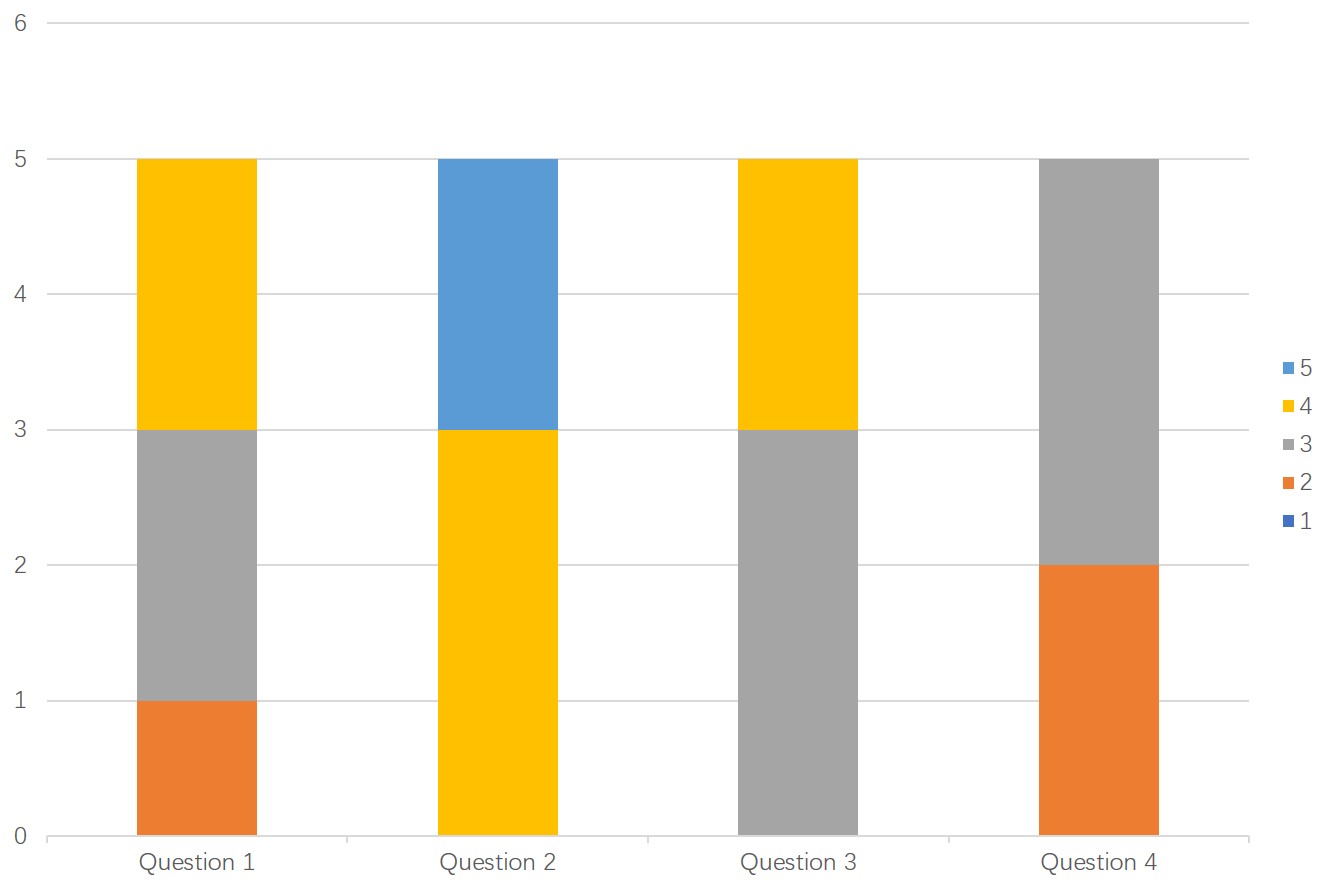

Supplement: Supplementary file 1 [file Image_1.jpg]
